# Genetic testing for susceptibility to major depressive disorder: a review of the behavioural repercussions of disclosing test results

**DOI:** 10.1097/YPG.0000000000000396

**Published:** 2025-04-23

**Authors:** Duru Kaya, Ulliana Savitskaya, Nicole Bloom

**Affiliations:** a Division of Psychology and Language Sciences, Faculty of Brain Sciences; b Division of Medicine, Faculty of Medical Sciences, University College London; c Department of Psychological Medicine, Institute of Psychiatry, Psychology & Neuroscience, King’s College London, London, UK

**Keywords:** *5-HTT*, depression, genetic testing, gene–environment, MDD, patient experience, psychoeducation

## Abstract

Predictive genetic testing for major depressive disorder (MDD) has become a widespread technological advancement to aid the process of early diagnosis and treatment selection. Despite these tests’ growing accessibility to the public, scant attention has been given to the behavioural changes that test-takers experience in response to undergoing the procedure and learning about their predisposition to MDD. The current paper aimed to be the first literature review to compile and evaluate the existing evidence demonstrating both the desirable and potentially harmful psychological responses following these tests. Studies portray a complicated picture, including desirable changes in the domains of felt stigma, lifestyle habits, and beliefs in treatment efficacy; as well as noteworthy deteriorations in perceived agency, fatalistic thoughts, and negativity bias in retrospective memory. In light of these findings, our review concludes that clear psychoeducation before testing is crucial to ensure that behavioural changes are predominantly beneficial for test-takers.

## Introduction

Psychiatric genetics has experienced a great leap forward with the recent establishment of the Human Genome Project, which illuminated the role of genetics in the aetiology and development of several mental health disorders (Cowan *et al*., 2002). Within the last 20 years, prevention science has revealed robust evidence that an interplay of genetic and environmental factors was crucial for the onset of various psychiatric disorders, including antisocial behaviour (Tuvblad *et al*., 2006), attention-deficit hyperactivity disorder (Sonuga-Barke *et al.*, 2009), and violence and aggression (Frazzetto *et al.*, 2007); however, perhaps one of the most substantial advances was achieved in understanding the epigenetics of major depressive disorder (MDD) and its diagnostic measures (Peña and Nestler, 2018).

The genetic influences involved in MDD were initially investigated via twin and adoption studies, which yielded an overall heritability rate of 40–50% (Sullivan *et al*., 2000). For instance, the twin study by Kendler *et al.* (2006) found the concordance rate of depression to be 0.44 in female monozygotic twins and 0.31 in male monozygotic twins, which were both higher than their dizygotic counterparts (0.16 and 0.11, respectively). Importantly, researchers found no significant correlation between the number of years the twins had spent living together and their symptoms of lifetime major depression, hence demonstrating the evident role of the genetic make-up.

With recent technological advancements in genomics, researchers have identified specific genes that could amplify one’s predisposition to MDD onset. An influential finding was provided by Caspi *et al.* (2003), who investigated the serotonin transporter gene (*SLC6A4*) as a candidate. *5-HTT* is a serotonin transporter protein coded by *SLC6A4*, which is responsible for regulating the reuptake of serotonin from the synaptic cleft (Goodman *et al.*, 1975). A polymorphism in *SLC6A4* may form a shorter allele (*s*), which codes for stronger *5-HTT* proteins and leaves smaller concentrations of serotonin in the synapse (Lesch *et al.*, 1996). Examining the consequences of this gene mutation, Caspi *et al*. (2003) found that individuals with one or more *s* alleles displayed more symptoms of MDD compared with those with the long allele (*l*) in response to stressful life events. This effect was stronger if individuals with the *s*/*l* or *s*/*s* genotype had experienced more than two distressing events, but a gene–environment interaction (G*x*E) was necessary for *SLC6A4* to be expressed and give rise to depressive symptoms.

Although currently subject to some scrutiny (Risch *et al.*, 2009), the findings of Caspi *et al*. (2003) were prominent in the integration of genetic risk markers of MDD into prevention science – most commonly known in the form of genetic test kits. It is estimated that more than 26 million people have taken direct-to-consumer genetic tests from ancestry and health companies during 2019, with numbers predicted to have increased in the following years (Regalado, 2019). These tests have the potential to aid preventive procedures, such that researchers and psychiatrists can more efficiently pinpoint individuals with the greatest risk of developing depression, swiftly directing them to the appropriate treatments (Wallace, 2004).

Despite these tests’ increasing popularity in both medical and nonclinical settings, the current literature demonstrates limited preliminary evidence showing the psychological repercussions of disclosing genetic susceptibility results to patients with MDD. Largely a by-product of the test’s novelty, most of the ethics discussions about the unintended behavioural changes of this instrument are restricted to opinion articles, requiring further empirical investigations to support their speculations.

These concerns are frequently centred around ‘genetic determinism’, which is the erroneous belief that all behaviours are inherently, and immutably, moderated by the genetic composition, rather than the external environment (Condit, 2011). Several studies indicate that the attribution of mental health disorders to biological factors is a leading precursor of ‘prognostic pessimism’, which is the hopelessness that individuals feel about their ability to prevent or cure a mental health disorder upon discovering a genetic predisposition (Deacon and Baird, 2009). Hence, test-takers who learn they have a biological susceptibility for MDD may lose faith in treatment altogether, fall into excessive worries about experiencing symptoms, and therefore engage in unhealthy behaviours that ultimately increase their likelihood of developing depression (Newson, 2009; Bortolotti and Widdows, 2011; Lawrie *et al.*, 2019; Ahn and Perricone, 2023).

As such unanticipated effects could have critical implications for the development of MDD and the efficacy of its treatment, it is crucial for clinicians and researchers to be able to conduct a well-informed cost- and benefit analysis before deciding on the test’s administration. Addressing this need, the current paper aimed to be the first literature review to compile the evidence demonstrating the behavioural changes that follow MDD genetic testing, discussing the clinical implications of both the desirable and possibly harmful psychological consequences for test-takers.

## Favourable psychological responses

### Part I: psychoeducation reduces stigma and increases perceived agency

One of the biggest concerns for administering genetic tests for MDD is that patients may struggle to understand the complex biochemical nature of the disorder and the meaning of the test results. As outlined in the introduction, a result of *s*/*s* genotype only indicates a high risk if the individual is exposed to stressful life events (Caspi *et al*., 2003). Even then, the test conveys a chance of vulnerability based on a small portion of the whole genome, rather than an inevitable fate. While this distinction may be difficult to assimilate compared with the intuitive appeal of genetic determinism, various findings propose that patients may be more responsive to the biochemical explanation than they are given credit for.

In one of the first qualitative investigations, Schreiber and Hartrick (2002) interviewed patients who had received medical attention for their depression within the past 5 years, which included receiving psychoeducation that delineated the genetic basis of their MDD. A key finding was that all participants fully embraced the biochemical explanation after being debriefed by their family physician or psychiatrist, even reconceptualising any personal experiences that contrasted with this neurological framework. This challenges the claim that patients may be disheartened by the complex genetic background of depression that is laid out to them during genetic testing because participants were able to revise their previous perspectives and describe their MDD with correct medical terminology. Schreiber and Hartrick (2002) also reported that a genetic understanding of depression helped patients mitigate the psychological burden caused by social stigma. Before being debriefed, participants described that they faced discrimination solely on the grounds of having MDD and felt ashamed for not being productive enough. The biogenetic understanding was employed to alleviate their self-induced stigma, helping them place less blame on themselves and feel relieved that their condition was not caused by their own insufficiency.

Similar findings were obtained by Lebowitz *et al*. (2013), who showed symptomatic individuals a psychoeducational video about the malleability of gene expression in MDD. Participants were then asked to write a letter to a hypothetical depressed individual, where they had to refer to the information they learned during psychoeducation to change this individual’s perception of depression. Although endorsing genetic factors as the cause of depression led patients to expect their symptoms to last longer (*β* = 0.18, *P* = 0.01) and decreased perceived odds of recovery (*β* = 0.15, *P* = 0.02), such feelings of prognostic pessimism were effectively reduced by a psychoeducational intervention. Being informed of the malleability of genetic factors significantly decreased patients’ expected symptom duration [*F*(2189) = 3.10, *P* < 0.05], increased their perceived likelihood of recovery [*F*(2187) = 5.31, *P* < 0.01], improved their confidence in managing future depressive episodes [*F*(2189) = 15.15, *P* < 0.01], and decreased their hopelessness [*F*(2175) = 5.67, *P* < 0.01]. This suggests that the psychoeducation provided during genetic testing for MDD susceptibility can mitigate the negative behavioural repercussions of the process, furthermore promoting desirable psychological changes in test-takers.

Building on these findings, Lebowitz and Ahn (2015) tested whether the beneficial behavioural changes of psychoeducation were long-lasting. Similar to the procedure of Lebowitz *et al*. (2013), symptomatic individuals were first shown a psychoeducational video about the malleability of gene expression in MDD and were later administered the negative mood regulation scale (Catanzaro and Mearns, 1990; Kemp *et al.*, 2014), and finally asked to estimate the duration of their depression. Six weeks later, participants were assigned the same questionnaires. The findings depicted that a brief psychoeducation on the malleability of genetic factors could significantly increase people’s perceived agency in managing depressive episodes [*F*(1450) = 24.90, *P* < 0.001], as well as decreasing prognostic pessimism [*F*(1449) = 10.49, *P* = 0.001]. Importantly, 6 weeks after the administration of the psychoeducational video, the increase in perceived agency [*F*(1250) = 10.90, *P* = 0.001] and decrease in prognostic pessimism [*F*(1250) = 4.07, *P* < 0.05] remained significant.

### Part II: test-takers’ perceived impact of genetic testing: expectations for early intervention, lifestyle changes, and reduced self-blame

Although Schreiber and Hartrick (2002), Lebowitz *et al*. (2013), and Lebowitz and Ahn (2015) provide useful insights into how symptomatic individuals might respond to the complicated neurobiological information during psychoeducation, another aspect of this debate concerns how patients respond to learning their genetic test results.

To investigate this, Wilhelm *et al.* (2009) recruited participants from a previous study about G*x*E interaction in depression’s onset (Wilhelm *et al.*, 2006), during which they consented to an analysis of their genetic material and were informed about *5-HTT* and the experiment by Caspi *et al*. (2003). Participants were offered to learn their genotype results, followed by a discussion of their perceived benefits and limitations related to MDD genotyping. In 2-week and 3-month follow-ups, researchers additionally assessed perceived future risk for developing depression, test-related distress, positive experiences, and satisfaction with the decision to learn results. Results revealed that 66% of participants requested to learn their results, indicating a mainly positive attitude towards genetic testing for MDD susceptibility. The majority of test-takers reported feeling pleased that they had learned the test outcome (92%), while none regretted receiving their result. The most frequently reported benefits were the test’s potential to enable early intervention (84%), prevent the onset of depression (83%), and help patients with the *s*/*s* or *s*/*l* genotype avoid stressors (77%). This finding therefore challenges concerns about prognostic pessimism, as it indicates that test-takers are able to appreciate the preventive potential of genetic testing for MDD and the efficacy of coping strategies to minimise their risk.

Nevertheless, given that the sample was obtained from a previous study investigating genetic vulnerability to depression, participants might have been more educated and inclined to assimilate a genetic explanation for MDD, hence depicting a more positive approach to testing than the general population. Addressing this limitation, Wilde *et al.* (2010) examined attitudes towards predictive genetic testing for depression among the nonclinical population. Before conducting interviews, participants were explained the process of genotyping for *5-HTT* polymorphism and the elevated risk of developing depression in the case of the *s*/*s* genotype. Participants were coded as either ‘affected’ (i.e. history of mental health disorders) or ‘unaffected’. Consistent with previous findings, the majority of the sample (67%) reported being interested in taking the test. During the interviews, several affected patients stated that learning about one’s genetic susceptibility for MDD would help validate their depression as a physical illness, diminishing the stress of social stigma and relieving them from the associated shame. Moreover, both affected and unaffected participants stated that the genetic tests for MDD predisposition would be highly beneficial for those with a family history of depression, allowing them to seek early help. Lastly, learning the test outcome was believed to help people adopt new behaviours to prevent the development or reduce the severity of MDD.

The preliminary findings of Wilde *et al*. (2010) were then replicated with a larger sample from the nonclinical general population by Wilde *et al.* (2011a), where researchers assessed community interest in genetic testing for MDD using a population-based survey. As with previous studies, there was a high participation rate (63%), indicating that the general population had a positive outlook on the test. In the case of getting a higher-than-average risk result, 85.7% of the sample stated they would be motivated to start receiving psychological help, reporting that they would integrate stress-reducing behaviours into their lives (83.2%), be able to distinguish the most effective treatment plan based on their genotype (82.2%) and validate their depression as a physical illness with genetic background (81.7%). Another perceived benefit was that receiving a lower-than-average risk result would alleviate any suspicions and provide test-takers with peace of mind (81%). Importantly, only 46.6% of the sample stated that the knowledge of having a higher susceptibility to MDD would prompt the development of a mental illness, suggesting that individuals may be less prone to prognostic pessimism compared with their strong endorsement of the benefits of the test.

Another qualitative study by Laegsgaard *et al.* (2010) recruited depressed patients with a family history of MDD from ongoing genetics studies at the Centre for Psychiatric Research, Aarhus University Hospital (Laegsgaard and Mors, 2008), and the Depression Network study (Farmer *et al.*, 2004). Researchers conducted interviews that explored how participating in a genetic analysis of MDD affected patients and their family’s views on depression and its stigma. Most participants reported altruistic motives for undergoing the procedure, believing that it made their suffering meaningful by helping future generations. Others reported wanting to feel more equipped to overcome depression before it progressed. MDD genotyping also increased patients’ family’s acceptance of the disorder. Participants reported that they felt more comfortable discussing their experiences of depression, finding it easier to seek help from medical professionals and family members. It was also stated that the genetic perspective they gained during the process legitimised their depression, which provided them relief and reduced their feelings of shame and guilt.

Lastly, Wilde *et al.* (2011b) conducted structured interviews with individuals from the nonclinical general population, aiming to assess the preventive behaviours they would integrate into their lives if they learnt they had a high genetic susceptibility to MDD. Results showed that participants who perceived their risk to be higher were more willing to start therapies or courses to acquire effective coping strategies (80%, *β* = 0.12, *P* = 0.001). These participants were also more likely to adopt new stress-managing behaviours (84%, *β* = 0.07, *P* = 0.029), hence indicating that genetic testing for MDD could be a motivating factor for at-risk individuals. Those who perceived the G*x*E interaction as a causal mechanism behind MDD were also more willing to participate in therapies or courses (*β* = 0.12, *P* = 0.017), employ stress-moderating behaviours (*β* = 0.10, *P* = 0.002), and teach their children how to cope with stress (92%, *β* = 0.16, *P* = 0.001). These findings underline the importance of psychoeducation before testing, as participants’ willingness to employ preventive behaviours appears to stem from an appreciation for the influence of environmental factors.

To summarise, the majority of clinical and nonclinical populations appear to have a positive outlook on the administration of genetic tests to determine susceptibility to MDD, with many being willing to take the test and learn their own genetic risk. During discussions of both the positive and negative behavioural repercussions of learning the test results, individuals frequently report that they would expect psychologically beneficial changes, such as an increased motivation to start therapies earlier, adopting healthy lifestyle changes, learning new stress-coping strategies, obtaining a piece of mind by reducing ambiguity, and feeling less guilt and shame for having depression.

Administering psychoeducation alongside the test also appears to have a crucial role in ensuring that the behavioural repercussions are positive. Unlike common expectations about prognostic pessimism, most participants seem to find the genetic explanations of MDD relieving, as it provides a biological disorder status to their depression and helps mitigate the blame placed on the individual. A clear explanation of the role of genetic factors in the development of depression appears integral for increasing patients’ perceived agency, hopefulness, and beliefs in treatment efficacy, as well as diminishing the pessimistic beliefs that may emerge after MDD genotyping. The main findings discussed in this section are summarised in Table 1.

**Table 1:** Summary of the key findings in the ‘favourable psychological responses’ chapter

Study	Key findings
Schreiber and Hartrick (2002)	Patients revised their understanding of MDD as a disorder with a genetic basis after psychoeducation, which provided relief, mitigated the stress of stigma, and reduced shame and guilt
Lebowitz *et al*. (2013)	Psychoeducation about the genetics of MDD reduced prognostic pessimism in symptomatic individuals, decreased perceived duration of depression, amplified perceived odds of recovery, increased agency, and decreased hopelessness
Lebowitz and Ahn (2015)	Psychoeducation about gene expression increased symptomatic individuals’ perceived agency and decreased prognostic pessimism, which continued up to 6 weeks
Wilhelm *et al*. (2009)	The majority of test-takers felt pleased to take the test; none regretted their participation. Reported benefits of the test were the enabling early intervention, preventing the first onset of depression, and avoiding stressors by employing healthy lifestyle changes
Wilde *et al*. (2010)	Perceived benefits were feeling reassured that depression was a neurobiological disorder, lower levels of stress and shame, possibilities of early diagnosis and prevention, and the adoption of healthy habits to mitigate symptoms
Wilde *et al*. (2011a)	The majority of participants reported that a high-risk result would motivate them to start therapies, adopt new behaviours to cope with stress, enable the selection of the most effective treatment, and validate their depression as a biological disorder. A low-risk result was also perceived beneficial to eliminate speculations
Laegsgaard *et al*. (2010)	Participants believed that MDD genotyping made their suffering more meaningful. They felt more prepared to mitigate symptoms, more confident discussing symptoms, and more comfortable asking for help. Genetic analysis increased the acceptance of depression in participants’ families
Wilde *et al*. (2011b)	In the case of a high-risk test result, individuals with a greater perceived risk were more willing to start therapies or courses and employ behaviours to cope with stress. Those who endorsed the importance of G*x*E in MDD were more likely to start therapy, adopt stress-reducing behaviours, and teach their children to cope with stress.

G*x*E, gene–environment interaction; MDD, major depressive disorder.

## Unfavourable psychological responses

### Part I: psychoeducation may lower treatment expectations and perceived likelihood of recovery

While the majority of findings support the benefits of psychoeducation in mitigating concerns about prognostic pessimism, there also exists contradictory evidence that should be evaluated when prescribing these tests. For instance, Schroder *et al.* (2020) examined how depressed patients’ views on treatment would be affected by a genetic understanding of their depression. Researchers recruited patients seeking treatment for MDD at a hospital, who were given the Beliefs About Cause of Depression Questionnaire (France *et al*., 2007), and the Credibility/Expectancy Questionnaire (Devilly and Borkovec, 2000). Results revealed that for patients with elevated depressive symptoms, endorsing the biogenetic causes for MDD was associated with poorer expectancy (*b* = −0.12, *P* = 0.0003) and credibility of treatment (*b* = −0.09, *P* = 0.004). This suggests that adoption of the biogenetic approach during psychoeducation has a risk of decreasing patients’ faith in recovery, thereby jeopardising their morale and progress in therapy (Rutherford *et al*., 2010).

### Part II: test-takers’ perceived negative impact of genetic testing: concerns about prognostic pessimism, discrimination, and memory biases

As with most psychological phenomena, the behavioural changes observed after going through genetic testing for MDD tend to be both positive and negative. Hence, most studies cannot conclude changes in one direction. This was also the case in the study by Wilhelm *et al.* (2009), previously described under ‘favourable psychological responses’. In addition to their aforementioned findings, Wilhelm *et al*. (2009) revealed that participants who underwent genetic testing for MDD experienced several concerns about the procedure and the test outcome. The most frequently reported concern among test-takers was that a high-susceptibility result could lead to discrimination by future employers (72%) or insurance companies (73%). Participants also reported having worries that being identified as high susceptibility could lead to feeling more stressed, depressed, or vulnerable (63%). Furthermore, test-takers with the *s*/*s* genotype result reported greater perceived lifetime risk than their *s*/*l*, *l*/*l*, and no-test counterparts, and experienced more distress. Those with the *s*/*s* genotype continued to exhibit significantly higher perceived lifetime risk and stress levels at the 2-week (*x*^2^ = 11.5, *P* = 0.003) and 3-month follow-ups (*x*^2^ = 13.0, *P* = 0.001).

Similar findings were observed in the previously described study by Wilde *et al.* (2010), which investigated attitudes towards predictive genetic testing for MDD among the general population. Some participants felt concerned that the confidentiality of their test results could not be guaranteed, risking discrimination by insurance companies and employers. Many also believed that they would develop fatalistic thoughts in the case of receiving a high-susceptibility test result, believing that they would eventually spiral into excessive worries and self-fulfilling behaviours. Several participants also worried that predictive genetic testing for depression would increase social stigma for at-risk individuals.

The study by Laegsgaard *et al.* (2010) also provided similar results in individuals with a personal and family history of depression. After going through MDD genotyping, some participants viewed depression as a condition that could not be prevented. Further evidencing this prognosis pessimism, some stated that the test would destroy all hopes of agency, preventing any possibility of improving one’s life. Although less prevalent than the reported positive changes, undergoing genetic analyses for MDD amplified pessimistic beliefs about the course of depressive symptoms for some test-takers.

Providing converging evidence, Lebowitz and Ahn (2018) examined the behavioural repercussions of genetic testing for MDD by revealing false results. Participants from the nonclinical population were randomly assigned a gene-presence, gene-absence, or inconclusive result after taking a sham genetic test for MDD. Those informed of a gene-presence felt they were more likely to have or develop depression than those in the gene-absent condition [*t*(163) = 5.22, *P* < 0.001], despite both groups having at most ‘mild depression’ before the test. Furthermore, gene-present individuals felt significantly less able to manage future negative emotions than their gene-absent counterparts [*t*(110) = 2.16, *P* = 0.03]. These findings therefore indicate that the knowledge of high genetic susceptibility for MDD can make individuals more pessimistic about their future symptoms and less confident about their agency; however, results also showed that a short psychoeducational video intervention was effective in mitigating such effects of prognostic pessimism [*t*(105) = 4.67, *P* < 0.001].

While this reiterates the importance of psychoeducation in genetic testing for MDD, a recent series of studies by Lebowitz and Ahn have brought attention to a more resilient behavioural change – a negativity bias in the recollection of depressive symptoms. In the study by Ahn *et al*. (2020), participants were asked to read a vignette about the symptoms of a depressed person, and a second vignette that explained the similar, but more severe, symptoms of another individual. When asked to recite the information, participants were more likely to remember the elevated symptoms from the second vignette as belonging to the first patient, if they were previously told that the first patient had a genetic predisposition to MDD [*F*(1522) = 4.96, *P* = 0.026]. This suggests that the knowledge of having a genetic susceptibility may influence people’s memory of past symptoms, misleadingly increasing their severity.

In addition, another study by Lebowitz and Ahn (2017) also demonstrated that a negativity bias in memory could be observed for participants’ recollection of their own MDD symptoms. Researchers administered mock direct-to-consumer genetic tests for depression, where participants were randomly assigned into a condition: gene-absent, gene-present, gene-present/intervention (where participants were later shown a psychoeducational video), or hypertension (where participants were told of a genetic susceptibility to hypertension). Participants then took the Beck Depression Inventory-II (BDI-II; Dozois, 2010), which measured their memory of exhibiting MDD symptoms in the past 2 weeks.

The gene-present group reported significantly greater BDI-II scores than the gene-absent condition [*F*(2426) = 6.71, *P* = 0.001], indicating that their false test result amplified their retroactive memory for depressive symptoms. Moreover, BDI-II scores were higher for those told of a gene-presence for depression compared with hypertension [*t*(579) = 2.79, *P* = 0.005], showing that the false recollection of symptoms was not simply the product of receiving unwelcome news. Lebowitz and Ahn (2017) also revealed that the BDI-II scores of the gene-present group (*n* = 141, *M* = 13.91) were significantly higher than the gene-absent condition (*n* = 143, *M* = 11.09) even after a psychoeducational video intervention (*P* = 0.036), which suggested that the negatively skewed reconstructions in test-takers’ memory were resistant to dissipation.

To summarise, by exacerbating an outlook of genetic determinism, predictive genetic tests for MDD susceptibility may have the potential to diminish an individual’s perceived ability at managing future depressive episodes. Such behavioural changes can reduce beliefs in treatment efficacy, which is crucial for adherence (Rutherford *et al*., 2010). Research exploring the effectiveness of psychoeducation in mitigating these negative repercussions is promising, the majority supporting its success; however, the finding that genetic tests can amplify one’s memory of symptoms is alarming, as the clinical diagnosis of depression heavily relies on the recollection of past depressive episodes. The implications of these findings will be explored further in the discussion section. The key findings described are summarised in Table 2.

**Table 2:** Summary of the key findings in the ‘unfavourable psychological responses’ chapter

Study	Key findings
Schroder *et al*. (2020)	In patients with more depressive symptoms, endorsing the biogenetic explanation of MDD correlated with lower recovery expectancy and poorer credibility of treatment
Wilhelm *et al*. (2009)	Participants worried testing could cause discrimination from employers and insurance companies, and make test-takers feel depressed. In baseline, 2-week, and 3-month follow-ups, those with the *s*/*s* genotype reported greater distress and perceived lifetime risk
Wilde *et al*. (2010)	Participants expressed concerns about social stigma and discrimination. Some believed they would form fatalistic thoughts if they received a high-susceptibility result, feel worried, and exhibit behaviours that eventually increase their risk
Laegsgaard *et al*. (2010)	MDD genotyping made some participants feel pessimistic about their future symptoms and decreased their perceived agency
Lebowitz and Ahn (2018)	Participants who received a high-risk test result believed they were more susceptible to have or develop depression than their gene-absent counterparts. Gene-present condition reported lower perceived agency than gene-absent groups
Ahn *et al*. (2020)	Participants were more likely to remember severe symptoms after being informed of a genetic susceptibility for MDD
Lebowitz and Ahn (2017)	The gene-present condition showed magnified retrospective memory for depressive symptoms. This negativity bias in memory did not dissipate with psychoeducation

MDD, major depressive disorder.

## Discussion

With genetic tests for MDD susceptibility becoming increasingly widespread, the current literature review aimed to provide an all-encompassing resource that clinicians, psychiatrists, and researchers could consult to review the possible psychological repercussions that might be exhibited by test-takers.

Our review suggests that learning the test outcome can be beneficial for the well-being of patients with MDD, as a higher-than-average risk result can provide tangible evidence for the biological basis of depression, hence mitigating the negative effects of social stigma. The alleviation of feelings of shame and guilt would be greatly beneficial for the treatment of MDD, as it would make it easier for patients to seek help sooner and build a social support network (Achterbergh *et al.*, 2020). Another important behavioural consequence is that the awareness of a genetic susceptibility to depression can encourage individuals to employ healthy lifestyle changes. Given that MDD is often characterised by a lack of motivation, this effect can be beneficial for patients or at-risk individuals to exercise more, eat healthier, socialise, and refrain from stressors, all of which accelerate the progress of treatment (George *et al.*, 1989; Wurtman, 2005; Eriksson and Gard, 2011; Khan and Khan, 2016). Lastly, a more accurate understanding of the G*x*E interaction in MDD and the knowledge of their own genotype can increase patients’ faith in recovery and drug efficacy, thereby ensuring that they commit to their treatment plan. This would address one of the most prevalent problems that clinicians face today, since patients can easily lose hope in finding the right medication, with premature dropout rates reaching 47% (Swift and Greenberg, 2012).

Nevertheless, our review also indicates that after receiving a high-risk result, test-takers can experience greater distress that persists in the long term, namely because of their concerns about the onset of their symptoms, workplace discrimination, and the confidentiality of their results from insurance companies. Such potential stressors are particularly notable, as both acute and chronic stress is linked to worsening symptoms and longer recovery times in depression’s treatment (Richter-Levin and Xu, 2018). Furthermore, genetic tests for MDD can cause some individuals to develop fatalistic thoughts about their agency. This behavioural repercussion should be evaluated carefully because feelings of prognostic pessimism can lead to a lack of motivation to take preventive measures, disengagement in treatment, and reduced medication efficacy (Rutherford *et al*., 2010).

A particularly concerning consequence of MDD genetic testing is its potential to distort patients’ memory of symptoms (Lebowitz and Ahn, 2017; Ahn *et al*., 2020). Given that psychiatric diagnoses heavily rely on patients’ retrospective self-reports of symptoms, memory biases elicited by genetic feedback could contribute to the overdiagnosis of MDD, as individuals may misremember their symptoms as being more severe than they were. Because of the memory-reliant nature of psychiatric assessment, such distortions in self-reported experiences may pose a risk for misdiagnoses and unnecessary treatment (Appelbaum and Benston, 2017). An incorrect diagnosis could risk the development of MDD in healthy individuals, causing them to experience elevated levels of stress, while simultaneously wasting the limited resources for treatment (Carey *et al.*, 2014). Moreover, despite psychoeducation’s success in mitigating other undesirable consequences of the procedure (e.g. Lebowitz *et al.*, 2013; Lebowitz and Ahn, 2015), the biased recall of depressive symptoms appears to be resistant to existing psychoeducational interventions. In light of these findings, a way for clinicians to mitigate negativity biases in memory could be to obtain patients’ symptom recollections prior to administering or disclosing genetic test results. In addition, clinical assessments may incorporate the observations of family members or friends to limit the influence of potential memory distortions in patients who believe themselves to be genetically susceptible to MDD. As genetic testing in psychiatry becomes more widespread, mental health practitioners must remain cautious about the potential diagnostic complications it may introduce and explore methods to mitigate its impact on patients’ beliefs in treatment efficacy and perceptions of past experiences. Psychoeducation appears to be a promising intervention, although further research is needed to improve its influence on negatively biased symptom memories.

Alternatively, a more impactful application of genetic testing for depression may lie in guiding individualised treatment selection rather than risk prediction. Traditionally, the prescription of antidepressants has been following a trial-and-error approach, with suboptimal response and remission rates (Thase *et al.*, 2005; Leucht *et al.*, 2009). By contrast, the emerging field of precision medicine utilises pharmacogenetics to enhance psychiatric prescriptions by tailoring drug selection and dosage to a patient’s genetics and environmental circumstances (Drew, 2016; Hodson, 2016). Indeed, recent tools for aiding clinical decisions employ pharmacogenetic tests to detect genetic variations that may be linked to antidepressant treatment responsiveness (Bousman and Hopwood, 2016). Common genes assessed in these tests include *CYP2C19* and *CYP2D6*, which encode enzymes that metabolise tricyclic antidepressants and the majority of selective serotonin reuptake inhibitors (Padmanabhan, 2014), hence constituting promising indicators of which antidepressants might be the most suitable for a given individual. Identifying the most effective treatment early is crucial, as antidepressants typically require 4–6 weeks to produce noticeable therapeutic effects (Jennings, 2018). Early selection of medication could furthermore reduce delays in symptom improvement, enhance patient morale, lower dropout rates, and ultimately lead to better long-term prognosis (Halfin, 2007). Nevertheless, despite its potential, pharmacogenetic testing in psychiatry lacks standardisation, as no two commercial tests assess the same combination of genetic markers, leading to inconsistencies in medication recommendations (Bousman *et al*., 2017). Furthermore, many pharmacogenetic test reports do not fully comply with established guidelines from the Clinical Pharmacogenetics Implementation Consortium and the Centres for Disease Control and Prevention, limiting their clinical reliability and practical implementation. Hence, the incorporation of pharmacogenetic tests into the selection of antidepressants is still in its infancy, requiring further research to address limitations in standardisation and compliance with pre-existing guidelines.

In summary, weighing the costs and benefits of genetic susceptibility testing for MDD, it is evident that these tests offer several advantages, including reducing stigma, encouraging positive lifestyle changes, and improving treatment adherence. These benefits can contribute to faster recovery, lower recurrence rates, and greater societal acceptance of MDD. Most of the potential psychological drawbacks – such as fatalistic thinking, distress, self-fulfilling prophecies, and false reassurance – can largely be mitigated through psychoeducation; however, memory distortion remains a persistent issue resistant to such interventions. A negatively biased recollection of symptoms may lead to overdiagnosis, incorrect treatment selection, and an increased risk of recurrence because of rumination and pre-existing negative cognitive schemas. Given these possible negative impacts, a more promising application of genetic testing for MDD may be its use for pharmacogenetic-guided drug selection to optimise treatment efficacy. Nevertheless, inconsistencies in the specific genetic markers analysed across different tests should be addressed to prevent discrepancies in metaboliser status predictions, which can otherwise lead to inconsistent medication recommendations.

### Limitations

Given the recency of these tests, as well as the preliminary nature of most studies, more empirical research is required to determine the permanency of the aforementioned behavioural changes summarised in this review. Because of ethical considerations, any deception regarding the genetic feedback for MDD predisposition cannot be continued for extended periods of time; however, it is crucial to observe the progress of these psychological changes, because test-takers may proceed to behave as if they already exhibit MDD, hence aggravating otherwise subclinical symptoms through a self-fulfilling prophecy (Turnwald *et al.*, 2019). As public access to genetic tests for MDD continues to increase, future studies should explore the long-term psychological effects of real-life genetic test results through correlational methods.

Furthermore, the existing research in this field has predominantly focussed on the behavioural repercussions of getting a higher-than-average risk result, as this would be more applicable to the clinical population. Nevertheless, with direct-to-consumer genetic testing kits increasing in popularity, it is expected that a large portion of test-takers will learn that they do not have an elevated predisposition to MDD. To our knowledge, only Wilde *et al’s*. (2011a) study has investigated participants’ inclination to fall into a false sense of reassurance, with 57.5% of participants reporting that they would consider themselves resilient to MDD regardless of any stressful life events. While this is a low percentage, more research is needed to conclude the generalisability of this belief of immunity that accompanies a *l*/*l* genotype result.

Another remaining gap in the literature concerns the discrepancy between the attitudes declared in interviews and the real-life behaviours participants would exhibit after taking a genetic test for MDD (FeldmanHall *et al.*, 2012). While qualitative studies are useful for identifying potential routes for research, they are nevertheless limited to hypothetical scenarios. This could indicate that participants may overestimate their constructive approach to a higher-than-average risk result, thereby not accurately representing the behavioural repercussions experienced by real-life test-takers.

Going beyond the possible effects of learning MDD predisposition results, future research should also pursue ways to mitigate these negative psychological changes and encourage the desirable attitudes. Our review demonstrates that psychoeducational interventions about the malleability of gene expression are significantly effective in reducing feelings of prognostic pessimism, as well as increasing perceived agency, hopefulness, beliefs in treatment efficacy, and motivation to employ preventive measures. Lebowitz and Ahn (2015) have also shown that these desirable behavioural changes persist up to six weeks. Hence, a clear psychoeducation that delineates the expression of genes in relation to the environment appears to be a promising method to safely conduct genetic tests for MDD.

Furthermore, interpreting genetic test results as indicating susceptibility to MDD should be done scrupulously, as such tests typically only investigate genes identified in previous candidate gene studies. This poses an issue for the field, as candidate gene studies have been difficult to replicate. More recent studies have suggested that the large effect sizes reported in previous investigations could instead be attributed to false positives (Border *et al*., 2019). Therefore, these limitations in replicability undermine the ability of genetic tests to accurately determine an individual’s susceptibility to MDD.

### Conclusion

The possible psychological consequences of genetic tests for depression is an ongoing debate, with studies depicting a relatively balanced picture of both desirable and undesirable repercussions for test-takers. The determining factor for whether patients experience the positive behavioural changes appears to be the psychoeducation that is provided with the test, although this has not been able to mitigate the negativity biases in memory yet. Given the test’s increasing accessibility to the public, more research should be conducted to illuminate the role of clinicians, researchers, and psychiatrists in articulating the necessity for a G*x*E interaction in MDD onset, as well as the correct interpretation of genotype results. While the field is only beginning to transition from preliminary qualitative findings to empirical studies, our existing understanding of genetic tests for MDD suggests that their impact depends largely on how results are conveyed before or after testing.

Given these findings, rather than relying on genetic risk prediction, which carries the risk of prognostic pessimism and psychological distress, genetic tests could perhaps be utilised in personalised medication selection. This alternative use has the potential to help clinicians prescribe the most suitable antidepressant at the right dose while avoiding the unintended negative consequences associated with susceptibility-focussed genetic testing. Nonetheless, future research should focus on improving the standardisation, accuracy, and regulatory oversight of pharmacological testing to enhance their clinical utility in psychiatric practice.
